# Molecular signaling in multiple myeloma: association of *RAS/RAF* mutations and MEK/ERK pathway activation

**DOI:** 10.1038/oncsis.2017.36

**Published:** 2017-05-15

**Authors:** J Xu, N Pfarr, V Endris, E K Mai, N H Md Hanafiah, N Lehners, R Penzel, W Weichert, A D Ho, P Schirmacher, H Goldschmidt, M Andrulis, M S Raab

**Affiliations:** 1 Max Eder Group Experimental Therapies for Hematologic Malignancies, Heidelberg University Hospital and German Cancer Research Center (DKFZ), Heidelberg, Germany; 2 Department of General Pathology, Institute of Pathology, Heidelberg University Hospital, Heidelberg, Germany; 3 Department of Internal Medicine V, Heidelberg University Hospital, Heidelberg, Germany; 4 National Center for Tumor Diseases (NCT), Heidelberg, Germany

## Abstract

Multiple myeloma (MM) is a plasma cell malignancy that is still considered to be incurable in most cases. A dominant mutation cluster has been identified in *RAS/RAF* genes, emphasizing the potential significance of RAS/RAF/MEK/ERK signaling as a therapeutic target. As yet, however, the clinical relevance of this finding is unclear as clinical responses to MEK inhibition in *RAS*-mutant MM have been mixed. We therefore assessed *RAS/RAF* mutation status and MEK/ERK pathway activation by both targeted sequencing and phospho-ERK immunohistochemistry in 180 tissue biopsies from 103 patients with newly diagnosed MM (NDMM) and 77 patients with relapsed/refractory MM (rrMM). We found a significant enrichment of *RAS/BRAF* mutations in rrMM compared to NDMM (*P*=0.011), which was mainly due to an increase of *NRAS* mutations (*P*=0.010). As expected, *BRAF* mutations were significantly associated with activated downstream signaling. However, only *KRAS* and not *NRAS* mutations were associated with pathway activation compared to *RAS/BRAF*^wt^ (*P*=0.030). More specifically, only *KRAS*^G12D^ and *BRAF*^V600E^ were consistently associated with ERK activation (*P*<0.001 and *P*=0.006, respectively). Taken together, these results suggest the need for a more specific stratification strategy consisting of both confirmation of protein-level pathway activation as well as detailed *RAS/RAF* mutation status to allow for a more precise and more effective application of targeted therapies, for example, with BRAF/MEK inhibitors in MM.

## Introduction

Multiple myeloma (MM) is a genetically highly heterogeneous disease. Despite tremendous improvement in response and survival rates due to novel agents and treatment strategies, MM is still considered to be incurable in the majority of patients.^1, 2^ Recent genomic studies suggest that MM is driven by mutations within the RAS signaling cascade. *KRAS*, *NRAS* and *BRAF* mutations are detectable in up to 50% of newly diagnosed MM patients.^3^ The incidence of mutations in most other genes is much lower, indicating the importance of the deregulation of key pathways, rather than mutations in single genes. Thus, the RAS/MEK/ERK pathway is currently believed to be activated in about half of MM cases and is therefore considered to be a major therapeutic target in MM like in many other cancers.^2, 4, 5, 6^ The activating *BRAF*^*V600E*^ mutation has been reported to be of therapeutic relevance and clinical trials exploring BRAF and/or MEK inhibition in this genetic setting are ongoing.^7, 8^ So far, treatment of *RAS/RAF* mutant MM with the MEK inhibitor, trametinib, resulted in only moderate response rates; however, some responding patients experienced durable remissions.^4^ This suggests the presence of varying degrees of dependency on MEK/ERK signaling in *RAS*-mutant MM.

Multiple downstream effector pathways have been reported to mediate RAS signaling, such as the RAF/MEK/ERK, PI3K/PDK1/AKT and TIAM1/RAC1 cascades, that regulate cell survival and proliferation, as well as cytoskeletal organization.^9^ In this study, we focused on the correlation between individual *RAS/BRAF* mutations, assessed by massive parallel sequencing technology, and actual MEK/ERK pathway activation, analyzed by immunohistochemistry for phosphorylated ERK1/2 as an activation marker in primary MM patient biopsies.^10^

## Results and discussion

### Patient characteristics

Formalin-fixed, paraffin-embedded bone marrow or extramedullary tissue samples were available from a total of 180 patients, including 103 newly diagnosed MM patients and 77 relapsed/refractory MM patients who relapsed from previous lines of therapy containing at least one immunomodulatory drug and one proteasome inhibitor. The majority of relapsed/refractory MM patients were refractory to at least one compound of either class. The median age and disease stage at diagnosis was comparable in newly diagnosed MM and relapsed/refractory MM patients (Table 1). Of note, to ensure consistent tissue preservation procedures, we included only patient samples from bone marrow trephines or soft tissue needle biopsies that were immediately preserved in 4% buffered formalin. The study was approved by the institutional review board (IRB206/2005 and 207/2005).

### RAS/RAF mutations in MM patients

**Figure 1:**
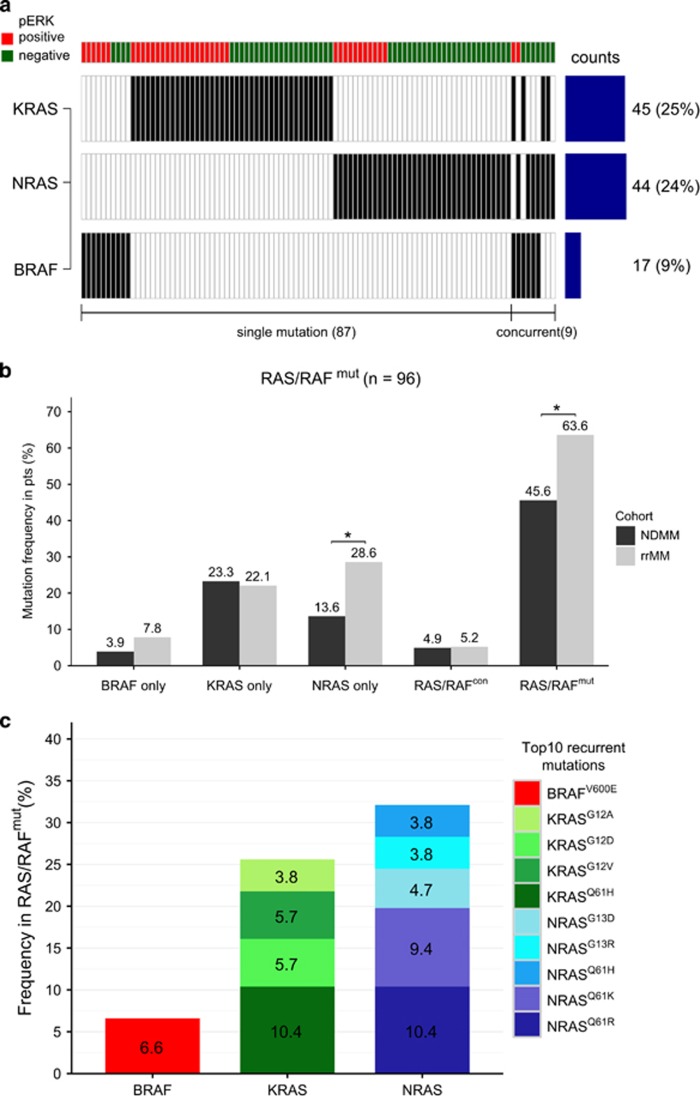
*RAS/RAF* mutation status and mutation frequency in MM. (**a**) A total of 96 out of 180 MM patients were identified with *KRAS* (45 cases), *NRAS* (44 cases) and *BRAF* mutations (17 cases) using a targeted panel (Ion AmpliSeq Cancer Hotspot panel v2, Ion Torrent/Thermo Fisher Scientific, Guilford, CT, USA), which covers *KRAS*/*NRAS* (exons 2, 3, 4), *HRAS* (exons 2, 3) and *BRAF* (exons 11,15). Targeted re-sequencing optimized for FFPE samples was performed as previously described.^21, 22^ In brief, data were analyzed with the Ion Torrent Suite Software (version 3.6, Ion Torrent/Thermo Fisher Scientific) against reference human genome hg19 and annotated using the CLC Genomics Workbench (CLC Bio/Qiagen, Aarhus, Denmark, version 6.5) with integrated information about nucleotide and amino-acid changes from RefSeq annotated genes, COSMIC (version 69, COSMIC database, Wellcome Trust Sanger Institute, Cambridge, UK) and dbSNP databases. Only variants with a minimum coverage >200 reads were considered. About 1600 × mean coverage for each amplicon was achieved. Overall, *RAS/RAF* mutations exhibited a mutually exclusive pattern, with 90.6% of the *RAS/RAF*^mut^ patients having single *KRAS*, *NRAS* or *BRAF* mutations, and only nine patients carried concurrent *RAS/RAF* mutations. The corresponding ERK activation status is shown on top, samples with moderate/strong immunohistochemical expression of pERK present in ⩾30% tumor cells were considered positive (red). (**b**) Comparison of *RAS/RAF* mutation frequencies in NDMM and rrMM cohorts. In general, *RAS/RAF*-mutant cases were significantly more frequent in rrMM compared to NDMM (*P<0.05). Notably, single *NRAS* mutations were significantly increased in rrMM, but not single *BRAF* or *KRAS* mutations. (**c**) Mutation frequencies in RAS/RAF^mut^ samples of the 10 most recurrent non-synonymous mutations are shown as stacked bar plots grouped by genes. FFPE, formalin-fixed, paraffin-embedded; NDMM, newly diagnosed MM; pERK, phosphorylated ERK1/2; rrMM, relapsed/refractory MM; SNP, single-nucleotide polymorphism.

Targeted sequencing identified 96/180 patients (53%) carrying at least one *RAS/BRAF* mutation with a nearly equal incidence in *NRAS* (24%) and *KRAS* (25%). *BRAF* mutations were detectable in 9% of patients. No *HRAS* mutations were found, consistent with previous studies.^2, 3, 4, 6, 11, 12^ The mutational spectrum was broad, including a total of nine types of non-synonymous substitutions in *BRAF*, 17 in *KRAS* and 12 in *NRAS*. In most cases, *RAS/RAF* mutations were mutually exclusive (90.6%). Only nine patients carried concurrent *RAS/RAF* mutations (Figure 1a).

Compared with newly diagnosed MM, the overall incidence of *RAS/BRAF* mutations was significantly higher in relapsed/refractory MM (*P*=0.011), mainly driven by a higher prevalence of *NRAS* mutations (*P*=0.010) (Figure 1b), suggesting that *NRAS* mutations may be involved in the development of drug resistance. This is supported by Mulligan *et al.*^13^ who observed that *NRAS*- but not *KRAS*-mutant MM had significantly lower response rates and a shorter time to progression following treatment with bortezomib monotherapy. However, Walker *et al.*^3^ found no association between *RAS* mutations and overall survival in a sample set from the National Cancer Research Institute Myeloma XI trial, a trial primarily based on immunomodulatory drug–cyclophosphamide combinations, indicating a treatment-specific resistance that might be overcome by combination therapies. One could also hypothesize that RAS-mutant clones are those that appear first at disease relapse, while the actual resistance might relate to an independent or co-operative mechanism. The RAS-mutant clones may not be the most aggressive ones, as patients surviving several lines of therapy are not likely to be those with the most aggressive forms of myeloma.

The most frequently affected codons of each gene were *BRAF* codon 600 (7/17, 41%), *NRAS* codon 61 (25/44, 57%) and *KRAS* codon 12 (19/45, 42%). The top 10 recurrently detected non-synonymous mutations were *KRAS*^Q61H^ (*n*=11), *NRAS*^Q61R^ (11), *NRAS*^Q61K^ (10), *BRAF*^V600E^ (7), *KRAS*^G12D^^/^^G12V^ (6 each), *NRAS*^G13D^ (5), *NRAS*^G13R/Q61H^ (4 each) and *KRAS*^G12A^ (4) (Figure 1c). In addition, we found a total of 10 patients carrying eight individual types of *BRAF*^non-V600E^ mutations within the kinase domain (CR3), including previously reported inactivating mutations G466V (*n*=1), G469E (1), D594A (1), D594G (2) and D594N (2), as well as activating mutations G469R (1), K601E (1); and one mutation with unknown function N581I (1) (Supplementary Table S1).^14^

### *RAS/RAF* mutations and MEK/ERK activation

**Figure 2:**
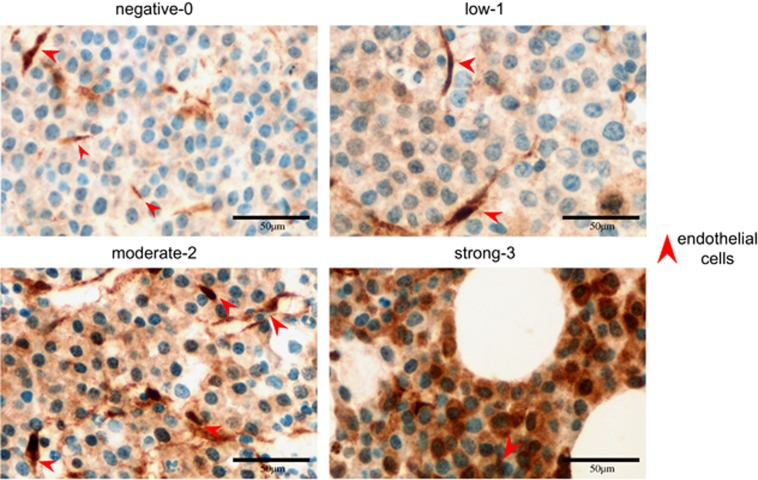
Evaluation of pERK expression in myeloma cells in bone marrow biopsies by IHC. The FFPE blocks were cut into 4–5 μm paraffin slides and stained with pERK antibody (#9101, Cell signaling, Danvers, MA, USA, 1:25, 24 min, 36 °C) using Ventana BenchMark Ultra Autostainer (Ventana Medical Systems/Roche, Tucson, AZ, USA) and OptiView DAB IHC detection Kit (Ventana Medical Systems/Roche). The staining protocol was optimized for pERK with CC1 (pH8.4) pretreatment for 64 min. For further information on effects of preservation protocols and controls, please refer to Supplementary Figure S2. The intensity of cytoplasmic staining of pERK in myeloma cells (i) was assessed in relation to endothelial cells (internal positive control cells for pERK, depicted by red arrow heads) and categorized into negative, low, moderate and strong (score 0–3), the percentage of positive tumor cells (*N*) was scored from 0 to 10, representing 0–100% of total assessable tumor cells. The cases with moderate/strong immunohistochemical expression of pERK present in ⩾30% tumor cells were classified as positive. FFPE, formalin-fixed, paraffin-embedded; IHC, immunohistochemistry; pERK, phosphorylated ERK1/2.

**Figure 3:**
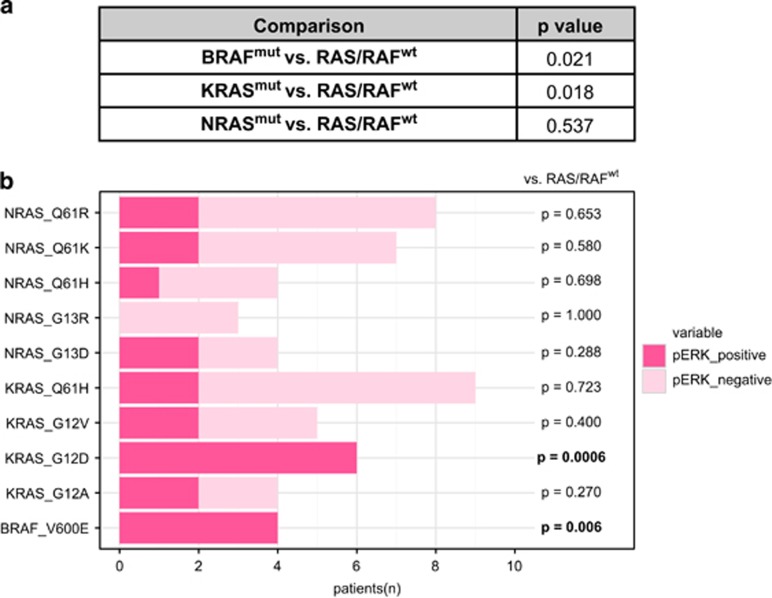
Correlation of RAS/RAF mutations with pERK expression. (**a**) *BRAF*^mut^, *KRAS*^mut^ and *NRAS*^mut^ cases were compared to *RAS/BRAF*^wt^ cases in relation to ERK activation. (**b**) Cases harboring one of the 10 most recurrent *RAS/RAF* mutations were tested against all *RAS/RAF*^wt^ cases for ERK activation status. A consistent association of ERK activation was only observed in cases with *KRAS*^G12D^ (*P*<0.001) and *BRAF*^V600E^ (*P*=0.006). All*P*-values were calculated using Fisher’s exact test. pERK, phosphorylated ERK1/2.

We then analyzed whether *RAS/RAF* mutations are associated with activation of the MEK/ERK signaling pathway, which is currently being investigated as a therapeutic target in MM. Concurrent mutations within the same pathway would impair a meaningful assessment of associations between genetic aberration and signaling activation. Therefore, only samples with a single *RAS* or *BRAF* mutation and an adjusted variant allele frequency (adjVAF)>10% were included into this analysis. While intracellular signaling can be activated by various upstream stimuli, mutations are likely to induce stronger downstream effects than physiologic conditions. Thus, only samples with phosphorylated ERK1/2 median/strong (intensity score >1) immunohistochemical expression being present in ⩾30% tumor cells (*n*=78) were considered as positive for the purpose of association studies (Figure 2). Overall, cases with mutant *RAS or BRAF* were significantly associated with activated MEK/ERK when compared to *RAS/BRAF*^wt^ (Figure 3a) (34%, compared to 52% in RAS/RAF-mutant samples; *P*=0.042). However, this relatively weak association already indicated that ERK activation might depend on the individual type of mutation within the respective gene. Therefore, we next analyzed recurrently found mutations separately for their association with downstream signaling.

Specifically, *BRAF*^V600E^ was consistently and significantly associated with strong ERK activation when compared to *RAS/BRAF*^wt^ (*P*=0.006) (Figure 3b). As mentioned previously, we also identified seven inactivating *BRAF* mutations of unknown relevance in MM. One mechanism by which these inactivating *BRAF* mutants may activate MEK/ERK signaling has been reported to involve paradoxical CRAF stimulation by the abrogation of negative feedback loops and promoting heterodimerization.^15, 16, 17^ Two of these cases were present as single *BRAF* mutation in a *RAS*^wt^ background. However, only one case with inactivating *BRAF*^D594N^ was detected in the majority of tumor cells (original variant allele frequency: 34%, tumor infiltration: 86.7%). This aberration was indeed associated with uniform ERK phosphorylation in 90% of the tumor cells.

In cases with *RAS* mutations, it is known from other cancer types that *KRAS* and *NRAS* have a distinct codon bias and that their codon-specific mutation frequencies differ in a disease-dependent manner, even though they represent structurally similar isoforms.^18^ We found *KRAS*^mut^ but not *NRAS*^mut^ to be associated with ERK activation when compared to *RAS/BRAF*^wt^ (*P*=0.018), suggesting that ERK activation may be dependent on the individual type of mutation rather than *RAS* gene mutation in general. We therefore sought to see whether there was an association of pathway activation with specific *KRAS* codon alterations. Samples with mutant *KRAS* in codons 12/13 versus codon 61 did not show a statistical significant association with ERK activation (*P*=0.085). All recurrent mutations were then tested individually against *RAS*^wt^*/BRAF*^wt^ samples (Figure 3b), as well as against other mutations within the same gene (Supplementary Figure S1): *KRAS*^G12D^ was associated with activated ERK in all cases (6/6) (*P*<0.001) (Figure 3b) and was more strongly associated with ERK activation than any other *KRAS* mutation (*P*=0.007) (Supplementary Figure S1). No significant association was observed between ERK activation and any of the other seven recurrent *RAS* mutations.

### Concurrent *RAS/RAF* mutations

In an additional nine cases, more than one mutation was found within *BRAF* and/or *RAS* genes. Six cases showed a co-occurrence of *BRAF/RAS* mutations in the same sample and three harbored two *RAS* mutations (2 × *KRAS/NRAS*, 1 × *NRAS/NRAS*). However, in only three samples both aberrations were detectable in the majority of MM cells, at a corrected allele frequency >40%, a level sufficient to be considered truly concurrent mutations in the same cell. These cases were positive for *KRAS*^Q61H^*/NRAS*^G13V^, *NRAS*^Q61K^*/NRAS*^I46M^ and *BRAF*^D594A^*/NRAS*^G12D^, respectively. In these samples, the cases with concurrent *RAS* mutations were not associated with significant activation of the MEK/ERK cascade. Interestingly, although representing only one single case, the inactivating *BRAF*^D594A^ together with activating *NRAS*^G12D^ was associated with strong phosphorylated ERK1/2 expression in nearly all tumor cells, in line with what has been reported from other tumor types on the cooperation of RAS signaling with impaired BRAF kinase activity conferred either by inactivating mutations^16, 17^ or by therapeutic BRAF kinase inhibitors such as vemurafenib or dabrafenib,^19, 20^ with the latter setting leading to resistance to these treatment approaches. This has also recently been shown in MM.^8^

Although intriguing, our observation will have to be proven experimentally or in a larger sample set. The biological and clinical relevance of the frequent subclonal *BRAF/RAS* mutations remains to be determined.

## Conclusion

In summary, this study on primary biopsies demonstrates for the first time that *BRAF/RAS* mutation status alone is not generally associated with MEK/ERK pathway activation, and may therefore not predict for therapeutic response to MEK inhibition in MM. In broader terms, the current paradigm that MM is substantially driven by activation of the MEK–ERK signaling cascade, due to the high prevalence of BRAF/RAS mutations, may not be true. Our data indicate that pathway-specific immunohistochemistry should be considered to assess pathway activation with the potential to inform future clinical trials of targeted therapies, for example, with, but likely not restricted to, BRAF/MEK inhibitors. Further validation of this concept of targeting activated pathways rather than potential marker mutations should be performed in the context of prospective clinical trials.

## Figures and Tables

**Table 1:** Patient characteristics and mutation status

*Characteristics/variables*	*Total (*n=*180)*	*NDMM (*n=*103)*	*rrMM (*n=*77)*
Male (*n*, %)	106 (59%)	67 (65%)	39 (51%)
Median age (yrs, range)	65 (31–86)	64 (31–86)	65 (36–85)
			
*Overall mutation status (*n*, %)*			
RAS/RAF^mut^	96 (53.3%)	47 (45.6%)	49 (63.6%)
RAS/RAF^wt^	84 (47%)	56 (42.8%)	28 (61.6%)
			
*RAS/RAF mutation carriers (*n*, %)*	Total (*n*=96)	NDMM (*n*=47)	rrMM (*n*=49)
Single RAS/RAF^mut^	87 (90.6%)	42 (89.4%)	45 (91.8%)
Concurrent RAS/RAF^mut^	9 (9.4%)	5 (4.9%)	4 (5.2%)

Abbreviations: NDMM, newly diagnosed multiple myeloma; rrMM, refractory/relapsed multiple myeloma.
