# Supplementary material for: Molecular signaling in multiple myeloma: association of RAS/RAF mutations and MEK/ERK pathway activation
Source: Oncogenesis. 2017 May 15;6(5):e337–. doi: 10.1038/oncsis.2017.36 (PMC5523069; doi:10.1038/oncsis.2017.36)
Supplement: Supplementary Information [file oncsis201736x1.pdf]

Supplementary Methods

Plasma cell purification

The peripheral blood mononuclear cell fraction was separated with Biocoll separating solution by density centrifugation (Biochrom GmbH, Berlin, Germany), and the plasma cells were separated using CD138-human MicroBeads and the MACS® Manual Cell Separator (Miltenyi Biotec, Bergisch Gladbach, Germany) following the instructions provided by the manufacturer. The purity of the cells was assessed by flow cytometry stained with FITC-mouse anti-human CD138 antibody (BD Biosciences, Heidelberg, Germany) with the average purity of 85%.

DNA extraction and QC

DNA was either extracted from FFPE tissue blocks (30 µm) or freshly purified tumor cells. For FFPE samples, automated DNA extraction procedure was applied using Maxwell FFPE Plus LEV DNA Purification Kit together with Maxwell 16 MDx Instrument LEV (AS3000) following manufacturer's instruction (Promega, Madison, WI, US). For fresh patient samples, the DNA was extracted from CD138 human MicroBeads purified cells (Miltenyi Biotec, Cologne, Germany) using QIAamp DNA Mini Kit (Qiagen, Hilden, Germany). DNA concentration was first determined by Qubit 2.0 Fluorometer using Qubit dsDNA HS Assay Kits (ThermoFisher Scientific, Darmstadt, Germany), and then further quantified by qPCR (TaqMan RNase P detection kit; ThermoFisher Scientific, Darmstadt, Germany).

Delay-of-fixation control experiments

The MM1.S human myeloma cells (7.5 mio cells/flask) were first starved for 24h in 1% FCS containing RPMI 1640 medium, then stimulated with 50 ng/ml human IL-6 (Sigma-Aldrich, Hamburg, Germany) for 10 minutes at 37°C. Equal numbers of non-stimulated cells served as controls. Cells were washed twice with PBS, incubated in PBS at RT for indicated time intervals (0min, 20min, 40min, 60min and 120min) before fixation in 4% formalin for 24h or protein extraction for immunoblotting, respectively. Cells in formalin were then embedded in HistoGel (ThermoFisher Scientific, Walldorf, Germany) according to manufacturer's instruction following standard FFPE sample embedding protocols. For each time point, non-stimulated, stimulated cells and pieces of normal human tonsils (24h fixation) were embedded together into one paraffin block. Each block was sectioned and stained with pERK antibody (#Cell signaling 9101) at the concentration of 1:25 (the same procedure as described in Figure 2 legend).

Supplementary Figure and Table Legend

Figure 1S. Association of pERK expression of the top 10 recurrent RAS/RAF mutations (left panel, magenta) and other mutations in respective gene (right panel, blue). Recurrent *RAS/RAF* mutations do not always correlate with ERK activation, the consistent association with MEK/ERK activation was only observed in cases with *KRAS*^{G12D} and *BRAF*^{V600E}. Each of the recurrent *RAS/RAF* mutations (magenta) was tested against other uncommon mutations (blue) within respective gene in relation to ERK action by Fisher's exact test. Compared to other *KRAS* mutations, *KRAS*^{G12D} is more likely to be associated with ERK activation (*P* = 0.007).

Figure S2. Delay-of-fixation control experiments. (**a**) Immunoblot of cell lysates from non-stimulated and IL6- stimulated MM1.S human myeloma cells at each delay-to-fixation time point. The membrane was incubated with primary antibody against pERK1/2 (#9101, 1:1000 in TBST with 5%BSA, Cell Signaling technology) and secondary antibody of horseradish peroxidase-conjugated goat anti-rabbit antibody (#sc-2004, 1:5000, Santa Cruz biotechnology, Heidelberg, Germany). Phosphorylation of ERK largely decreased over time mainly in non-stimulated cells with less pronounced decrease in stimulated cells. (**b**) IHC staining of non-stimulated and IL6- stimulated MM1.S cells from the same experiment. Cells were embedded into FFPE blocks and stained with the same anti-pERK antibody at the dilution of 1:25. Background ERK phosphorylation decreased rapidly if cells were not preserved immediately. Stimulation by IL-6 delayed decay of phosphorylation by approximately 40min. Of note, no significant phosphorylation of ERK was detected in plasma cells of tonsils or bone marrow form healthy controls.

Table 1S. Immunohistochemistry and targeted sequencing results of all samples. Explanations to abbreviated column names: PC%- plasma cell percentage (tumor infiltration rate) measured by CD138 staining of the FFPE sample (if DNA was extracted from the FFPE block) or flow cytometry of CD138 for sorted cells; oriVAF - original variant allele frequency in percentage; adjVAF – adjusted variant allele frequency in percentage; pERK_i - pERK intensity in plasma cells (range from 0-negative to 3-strongly positive); pERK_N - pERK positive tumor percentage (range from 0 – none, to 10 - positive in 100% tumor); pos_I1N3- pERK positivity based on cut-off of I = 1 and N =3. QCpERK- quality control score of pERK staining, only samples with strong, homogeneous pERK immunoreactivity in internal positive control cells (endothelial cells) – score 2 and 3 were eligible for correlation analysis.
