# Supplementary material for: Molecular signaling in multiple myeloma: association of RAS/RAF mutations and MEK/ERK pathway activation
Source: Oncogenesis. 2017 May 15;6(5):e337–. doi: 10.1038/oncsis.2017.36 (PMC5523069; doi:10.1038/oncsis.2017.36)
Supplement: Supplementary Table 1 [file oncsis201736x2.pdf]

ID	Age	Sex	cohort	material_NGS	PC%	Gene	AAchange	COSMIC.ID	Codon	Coverage	oriVAF	adjVAF	pERK_i	pERK_N	QCpERK
1	63	M	NDMM	Bone marrow	50	KDR	Q472H	149673	472	2173	49.7	49.7	1	7	3
1	63	M	NDMM	Bone marrow	50	KRAS	Q61P	551	61	3758	7.1	14.2	1	7	3
1	63	M	NDMM	Bone marrow	50	TP53	P72R	250061	72	1011	54.8	54.8	1	7	3
2	46	F	rrMM	Soft tissue	100	FLT3	N587S		587	603	85.9	85.9	2	8	1
2	46	F	rrMM	Soft tissue	100	KDR	Q472H	149673	472	1537	98.3	98.3	2	8	1
2	46	F	rrMM	Soft tissue	100	KIT	NA		NA	2969	42.6	42.6	2	8	1
2	46	F	rrMM	Soft tissue	100	NRAS	Q61R	584	61	3581	85.9	85.9	2	8	1
2	46	F	rrMM	Soft tissue	100	PIK3CA	I391M	328028	391	496	14.3	14.3	2	8	1
2	46	F	rrMM	Soft tissue	100	TP53	P72R	250061	72	2010	98.4	98.4	2	8	1
3	60	M	rrMM	Bone marrow	70	KDR	Q472H	149673	472	1182	49.8	49.8	0	0	2
3	60	M	rrMM	Bone marrow	70	TP53	P72R	250061	72	1015	72.3	72.3	0	0	2
4	69	M	rrMM	Bone marrow	30	KDR	Q472H	149673	472	952	97.9	97.9	1	3	1
5	36	M	rrMM	Soft tissue	100	ATM	F858L	21826	858	2904	49.2	49.2	1	3	1
5	36	M	rrMM	Soft tissue	100	NRAS	Q61K	580	61	3680	47.6	47.6	1	3	1
5	36	M	rrMM	Soft tissue	100	PIK3CA	I391M	328028	391	644	36.2	36.2	1	3	1
5	36	M	rrMM	Soft tissue	100	TP53	P72R	250061	72	1377	97.4	97.4	1	3	1
6	49	M	NDMM	Bone marrow	60	BRAF	G469E	461	469	1062	6.9	11.5	0	0	2
6	49	M	NDMM	Bone marrow	60	KDR	Q472H	149673	472	1542	50.9	50.9	0	0	2
6	49	M	NDMM	Bone marrow	60	NRAS	G12V	566	12	1414	7.4	12.3	0	0	2
6	49	M	NDMM	Bone marrow	60	TP53	P72R	250061	72	1130	98.2	98.2	0	0	2
7	68	F	rrMM	Soft tissue	100	KDR	Q472H	149673	472	375	5.6	5.6	1	1	2
7	68	F	rrMM	Soft tissue	100	KIT	M541L	28026	541	1535	48.7	48.7	1	1	2
7	68	F	rrMM	Soft tissue	100	NRAS	G13D	573	13	1114	39.5	39.5	1	1	2
7	68	F	rrMM	Soft tissue	100	TP53	P72R	250061	72	1029	54.5	54.5	1	1	2
8	68	M	NDMM	Bone marrow	30	KDR	Q472H	149673	472	1026	53.6	53.6	0	0	3
8	68	M	NDMM	Bone marrow	30	NRAS	G12V	566	12	2401	8.7	29	0	0	3
8	68	M	NDMM	Bone marrow	30	TP53	P72R	250061	72	554	97.2	97.2	0	0	3
9	61	M	NDMM	Bone marrow	90	KRAS	G13D	1140132, 532	13	1361	42.1	42.1	3	8	2
9	61	M	NDMM	Bone marrow	90	TP53	P72R	250061	72	184	52.3	52.3	3	8	2
10	67	F	rrMM	Bone marrow	100	NRAS	Q61R	584	61	2581	38.9	38.9	2	1	2
10	67	F	rrMM	Bone marrow	100	TP53	P72R	250061	72	3145	98.5	98.5	2	1	2
11	40	F	rrMM	Soft tissue	100	KDR	Q472H	149673	472	917	14.2	14.2	3	6	2
11	40	F	rrMM	Soft tissue	100	TP53	P72R	250061	72	402	98.3	98.3	3	6	2
11	40	F	rrMM	Soft tissue	100	VHL	R167W	14311	167	2744	45.1	45.1	3	6	2
12	61	M	NDMM	Bone marrow	10	KIT	M541L	28026	541	3736	49.3	49.3	0	0	3
12	61	M	NDMM	Bone marrow	10	TP53	P72R	250061	72	1740	98.5	98.5	0	0	3
13	65	F	NDMM	Bone marrow	80	KDR	Q472H	149673	472	1498	41.7	41.7	1	1	3
13	65	F	NDMM	Bone marrow	80	KRAS	G60R		60	4325	13.9	13.9	1	1	3
13	65	F	NDMM	Bone marrow	80	TP53	P72R	250061	72	1713	97.5	97.5	1	1	3
14	65	M	rrMM	Soft tissue	100	CDKN2A	NA	12484, 1314728, 753735, 753736	NA	1294	48.4	48.4	0	0	2

14	65	M	rrMM	Soft tissue	100	TP53	P72R	250061	72	2438	98.4	98.4	0	0	2
15	58	M	NDMM	Bone marrow	70	ATM	S1691R		1691	1660	56.8	56.8	0	0	2
15	58	M	NDMM	Bone marrow	70	TP53	P72R	250061	72	116	96.6	96.6	0	0	2
16	42	M	NDMM	Bone marrow	90	KDR	Q472H	149673	472	1564	49.9	49.9	2	2	2
16	42	M	NDMM	Bone marrow	90	TP53	P72R	250061	72	478	96.2	96.2	2	2	2
17	63	M	NDMM	Bone marrow	20	KDR	Q472H	149673	472	2300	49.8	49.8	0	0	2
17	63	M	NDMM	Bone marrow	20	TP53	P72R	250061	72	1491	95.1	95.1	0	0	2
18	86	M	NDMM	Soft tissue	100	BRAF	V600E	476	600	1137	44.7	44.7	0	0	1
18	86	M	NDMM	Soft tissue	100	TP53	P72R	250061	72	914	58	58	0	0	1
19	54	F	rrMM	Bone marrow	60	MET	N375S	710	375	1169	45.3	45.3	3	6	3
19	54	F	rrMM	Bone marrow	60	TP53	P72R	250061	72	656	56.6	56.6	3	6	3
20	69	M	NDMM	Soft tissue	100	KDR	Q472H	149673	472	1074	48.7	48.7	0	0	3
20	69	M	NDMM	Soft tissue	100	RET	S649L		649	1619	44.9	44.9	0	0	3
20	69	M	NDMM	Soft tissue	100	TP53	P72R	250061	72	1527	95.5	95.5	0	0	3
21	60	F	NDMM	Bone marrow	20	PIK3CA	I391M	328028	391	648	60.3	60.3	0	0	0
21	60	F	NDMM	Bone marrow	20	TP53	P72R	250061	72	534	52.2	52.2	0	0	0
22	62	M	NDMM	Bone marrow	60	JAK3	V722I	34213	722	1387	59.2	59.2	0	0	3
22	62	M	NDMM	Bone marrow	60	NRAS	Q61K	580	61	1890	16.8	16.8	0	0	3
22	62	M	NDMM	Bone marrow	60	TP53	P72R	250061	72	572	96.6	96.6	0	0	3
23	52	M	NDMM	Bone marrow	60	TP53	P72R	250061	72	505	53.8	53.8	0	0	3
24	55	M	NDMM	Bone marrow	30	BRAF	G466V	451	466	261	5	16.7	2	9	2
24	55	M	NDMM	Bone marrow	30	KRAS	Q61H	1135364, 554	61	969	16.5	16.5	2	9	2
24	55	M	NDMM	Bone marrow	30	TP53	P72R	250061	72	422	60.6	60.6	2	9	2
25	56	F	rrMM	Bone marrow	80	BRAF	V600E	476	600	422	44.1	44.1	3	4	3
25	56	F	rrMM	Bone marrow	80	PIK3CA	I391M	328028	391	345	53.8	53.8	3	4	3
25	56	F	rrMM	Bone marrow	80	TP53	P72R		72	413	49.8	49.8	3	4	3
26	57	M	NDMM	Bone marrow	60	KDR	Q472H	149673	472	1081	100	100	0	0	2
26	57	M	NDMM	Bone marrow	60	PIK3CA	I391M	328028	391	1130	47.2	47.2	0	0	2
26	57	M	NDMM	Bone marrow	60	TP53	P72R	250061	72	560	53.8	53.8	0	0	2
27	70	M	NDMM	Bone marrow	70	MET	N375S	710	375	682	38.7	38.7	0	0	3
27	70	M	NDMM	Bone marrow	70	TP53	P72R	250061	72	397	64.9	64.9	0	0	3
27	70	M	NDMM	Bone marrow	70	VHL	P81S	17721	81	609	50.5	50.5	0	0	3
28	74	F	NDMM	Bone marrow	100	FGFR3	C275Y		275	1278	18.1	18.1	1	1	3
28	74	F	NDMM	Bone marrow	100	KDR	Q472H	149673	472	659	51.7	51.7	1	1	3
28	74	F	NDMM	Bone marrow	100	TP53	P72R	250061	72	528	100	100	1	1	3
29	66	M	NDMM	Bone marrow	70	BRAF	N581I	26460	581	1727	24.1	24.1	1	1	3
29	66	M	NDMM	Bone marrow	70	PIK3CA	I391M	328028	391	1015	55.1	55.1	1	1	3
29	66	M	NDMM	Bone marrow	70	TP53	P72R	250061	72	563	97.4	97.4	1	1	3
30	71	F	NDMM	Bone marrow	80	NRAS	Q61R	584	61	2709	31.5	31.5	2	5	2
30	71	F	NDMM	Bone marrow	80	PIK3CA	I391M	328028	391	1361	52.9	52.9	2	5	2
30	71	F	NDMM	Bone marrow	80	TP53	P72R	250061	72	631	62.6	62.6	2	5	2

31	66	F	NDMM	Bone marrow	10	KDR	Q472H	149673	472	1166	53.5	53.5	0	0	3
31	66	F	NDMM	Bone marrow	10	NRAS	G13R	569	13	1364	5.6	56	0	0	3
31	66	F	NDMM	Bone marrow	10	TP53	P72R	250061	72	712	63.1	63.1	0	0	3
32	67	F	NDMM	Bone marrow	90	KRAS	G12A	1140134, 522	12	1874	19.8	19.8	3	8	2
32	67	F	NDMM	Bone marrow	90	TP53	P72R	250061	72	481	67.6	67.6	3	8	2
33	57	M	NDMM	Bone marrow	80	TP53	NA	10762, 707872, 707873, 707874, 707875	NA	2051	13.2	13.2	3	3	2
33	57	M	NDMM	Bone marrow	80	TP53	P72R	250061	72	709	65.1	65.1	3	3	2
34	64	M	NDMM	Bone marrow	50	KDR	Q472H	149673	472	1478	51.3	51.3	0	0	2
34	64	M	NDMM	Bone marrow	50	PIK3CA	I391M	328028	391	802	61.8	61.8	0	0	2
34	64	M	NDMM	Bone marrow	50	TP53	P72R	250061	72	782	63.7	63.7	0	0	2
35	67	M	rrMM	Soft tissue	100	IDH1	R132C	28747	132	2516	37.9	37.9	1	5	2
35	67	M	rrMM	Soft tissue	100	JAK3	V722I	34213	722	1311	50.2	50.2	1	5	2
35	67	M	rrMM	Soft tissue	100	KDR	Q472H	149673	472	1229	54	54	1	5	2
35	67	M	rrMM	Soft tissue	100	KIT	M541L	28026	541	3025	47.4	47.4	1	5	2
35	67	M	rrMM	Soft tissue	100	NRAS	Q61K	12730	61	2155	61.2	61.2	1	5	2
35	67	M	rrMM	Soft tissue	100	NRAS	I46M		46	2141	61.3	61.3	1	5	2
35	67	M	rrMM	Soft tissue	100	PIK3CA	I391M	328028	391	1109	54.8	54.8	1	5	2
35	67	M	rrMM	Soft tissue	100	TP53	P72R	250061	72	734	65	65	1	5	2
36	68	F	rrMM	Bone marrow	70	ERBB2	NA		NA	6219	23.4	23.4	1	1	2
36	68	F	rrMM	Bone marrow	70	NRAS	G13R	569	13	2537	26.2	26.2	1	1	2
36	68	F	rrMM	Bone marrow	70	PIK3CA	I391M	328028	391	1600	51.7	51.7	1	1	2
36	68	F	rrMM	Bone marrow	70	TP53	P72R	250061	72	1172	51.4	51.4	1	1	2
37	60	M	rrMM	Bone marrow	100	KRAS	Q61H	1135364, 554	61	1392	42.7	42.7	0	0	3
37	60	M	rrMM	Bone marrow	100	NRAS	G13V	574	13	1203	45.2	45.2	0	0	3
37	60	M	rrMM	Bone marrow	100	TP53	P72R	250061	72	2037	100	100	0	0	3
38	69	M	NDMM	Bone marrow	90	KDR	Q472H	149673	472	1927	50.2	50.2	1	1	2
38	69	M	NDMM	Bone marrow	90	PIK3CA	I391M	328028	391	1505	32.1	32.1	1	1	2
38	69	M	NDMM	Bone marrow	90	TP53	P72R	250061	72	779	96.7	96.7	1	1	2
39	65	F	NDMM	Bone marrow	40	KDR	Q472H	149673	472	1376	3.6	9	3	5	2
39	65	F	NDMM	Bone marrow	40	TP53	P72R	250061	72	1150	50.6	50.6	3	5	2
40	65	M	NDMM	Bone marrow	80	KDR	Q472H	149673	472	765	48.1	48.1	0	0	2
40	65	M	NDMM	Bone marrow	80	TP53	P72R	250061	72	294	98	98	0	0	2
41	69	M	NDMM	Bone marrow	30	KDR	Q472H	149673	472	1368	50	50	0	0	2
41	69	M	NDMM	Bone marrow	30	KIT	M541L	28026	541	2826	52.3	52.3	0	0	2
42	58	M	rrMM	Soft tissue	100	BRAF	D594A	1583010	594	1244	41.2	41.2	2	9	2
42	58	M	rrMM	Soft tissue	100	NRAS	G12D	564	12	1698	41.6	41.6	2	9	2
42	58	M	rrMM	Soft tissue	100	TP53	NA	11063, 1640834, 301402, 301403, 301404	NA	829	66.3	66.3	2	9	2
42	58	M	rrMM	Soft tissue	100	TP53	P72R	250061	72	263	98.1	98.1	2	9	2
43	60	M	NDMM	Bone marrow	15	KDR	Q472H	149673	472	1438	50.7	50.7	1	2	2
43	60	M	NDMM	Bone marrow	15	TP53	P72R	250061	72	744	98.4	98.4	1	2	2
44	53	M	NDMM	Bone marrow	80	KDR	Q472H	149673	472	412	54.3	54.3	1	1	2

44	53	M	NDMM	Bone marrow	80	KRAS	Q61H	1135364, 554	61	5203	6.9	8.6	1	1	2
44	53	M	NDMM	Bone marrow	80	TP53	P72R	250061	72	180	95.2	95.2	1	1	2
45	63	M	NDMM	Bone marrow	90	KIT	M541L	28026	541	4120	51.8	51.8	2	3	2
45	63	M	NDMM	Bone marrow	90	TP53	P72R	250061	72	1268	97.7	97.7	2	3	2
46	39	M	NDMM	Bone marrow	80	KDR	Q472H	149673	472	1115	50.9	50.9	0	0	3
46	39	M	NDMM	Bone marrow	80	PIK3CA	I391M	328028	391	652	58.6	58.6	0	0	3
46	39	M	NDMM	Bone marrow	80	TP53	P72R	250061	72	639	98.3	98.3	0	0	3
47	58	M	NDMM	Bone marrow	90	KRAS	G12D	1135366, 521	12	1429	33.3	33.3	3	8	2
48	51	M	NDMM	Bone marrow	70	TP53	NA	10662, 1640830, 99020, 99021, 99602	NA	2450	26	26	0	0	3
48	51	M	NDMM	Bone marrow	70	TP53	NA	10733, 117946, 117947, 117948, 117949, 1649390	NA	1215	26.1	26.1	0	0	3
48	51	M	NDMM	Bone marrow	70	TP53	P72R	250061	72	399	100	100	0	0	3
49	60	M	NDMM	Bone marrow	70	KDR	Q472H	149673	472	963	56.6	56.6	0	0	2
49	60	M	NDMM	Bone marrow	70	TP53	P72R	250061	72	629	96.1	96.1	0	0	2
50	79	F	NDMM	Bone marrow	80	ATM	A1309T	22507	1309	1896	41.3	41.3	3	8	2
50	79	F	NDMM	Bone marrow	80	KDR	Q472H	149673	472	913	64.4	64.4	3	8	2
50	79	F	NDMM	Bone marrow	80	KRAS	A146T	1165198, 19404	146	642	21.5	21.5	3	8	2
50	79	F	NDMM	Bone marrow	80	TP53	P72R	250061	72	638	97.2	97.2	3	8	2
51	74	M	NDMM	Bone marrow	75	FGFR3	NA	1539830, 724	NA	1209	53	53	1	3	3
51	74	M	NDMM	Bone marrow	75	KDR	Q472H	149673	472	939	50.8	50.8	1	3	3
51	74	M	NDMM	Bone marrow	75	KRAS	Q61H	1135364, 554	61	2619	11.2	11.2	1	3	3
51	74	M	NDMM	Bone marrow	75	PTPN11	F71L	13029	71	2250	12.2	12.2	1	3	3
51	74	M	NDMM	Bone marrow	75	TP53	C229*	45394	229	4189	12.2	12.2	1	3	3
51	74	M	NDMM	Bone marrow	75	TP53	P72R	250061	72	1773	92.7	92.7	1	3	3
52	69	F	NDMM	Bone marrow	65	BRAF	K601E	478	601	1496	5.2	8	0	0	2
52	69	F	NDMM	Bone marrow	65	BRAF	D594G	467	594	1502	5.4	8.3	0	0	2
52	69	F	NDMM	Bone marrow	65	MET	N375S	710	375	1262	48.5	48.5	0	0	2
52	69	F	NDMM	Bone marrow	65	NRAS	Q61R	584	61	4295	5.2	8	0	0	2
52	69	F	NDMM	Bone marrow	65	TP53	P72R	250061	72	663	64.7	64.7	0	0	2
53	80	M	NDMM	Bone marrow	60	KDR	Q472H	149673	472	1801	52.5	52.5	0	0	3
53	80	M	NDMM	Bone marrow	60	KRAS	G12A	1140134, 522	12	1893	17.1	17.1	0	0	3
53	80	M	NDMM	Bone marrow	60	TP53	P72R	250061	72	776	65.5	65.5	0	0	3
54	69	F	NDMM	Bone marrow	55	KDR	Q472H	149673	472	423	49.1	49.1	0	0	2
54	69	F	NDMM	Bone marrow	55	TP53	P72R	250061	72	428	95.7	95.7	0	0	2
55	68	M	NDMM	Bone marrow	90	KDR	Q472H	149673	472	1520	49.5	49.5	3	6	2
55	68	M	NDMM	Bone marrow	90	TP53	P72R	250061	72	730	100	100	3	6	2
56	69	M	NDMM	Bone marrow	40	KDR	Q472H	149673	472	874	53.9	53.9	1	1	2
56	69	M	NDMM	Bone marrow	40	MET	E168D	706	168	1763	47.7	47.7	1	1	2
56	69	M	NDMM	Bone marrow	40	NRAS	Q61K	580	61	1948	7.1	17.7	1	1	2
56	69	M	NDMM	Bone marrow	40	TP53	P72R	250061	72	298	53.6	53.6	1	1	2
57	60	F	NDMM	Bone marrow	90	ATM	G2695D	1351010, 1351011	2695	4292	32.5	32.5	0	0	0
57	60	F	NDMM	Bone marrow	90	KIT	M541L	28026	541	2976	50.9	50.9	0	0	0

57	60	F	NDMM	Bone marrow	90	TP53	P72R	250061	72	968	98.3	98.3	0	0	0
58	68	F	NDMM	Bone marrow	100	TP53	NA	121035, 121036, 121037, 1640833, 6932	NA	3484	82.2	82.2	0	0	3
58	68	F	NDMM	Bone marrow	100	TP53	P72R	250061	72	36	89.2	89.2	0	0	3
59	82	M	NDMM	Bone marrow	85	KDR	Q472H	149673	472	1068	51.2	51.2	1	1	0
59	82	M	NDMM	Bone marrow	85	TP53	P72R	250061	72	1073	55.9	55.9	1	1	0
60	86	F	NDMM	Bone marrow	90	STK11	F354L	21360	354	1189	38.6	38.6	0	0	3
60	86	F	NDMM	Bone marrow	90	TP53	NA		NA	1461	57.5	57.5	0	0	3
60	86	F	NDMM	Bone marrow	90	TP53	P72R	250061	72	420	21.3	21.3	0	0	3
61	83	M	NDMM	Bone marrow	20	KDR	Q472H	149673	472	686	51.2	51.2	2	1	3
61	83	M	NDMM	Bone marrow	20	PIK3CA	I391M	328028	391	485	53.2	53.2	2	1	3
61	83	M	NDMM	Bone marrow	20	TP53	P72R	250061	72	292	54.8	54.8	2	1	3
62	75	M	NDMM	Bone marrow	20	KDR	Q472H	149673	472	1234	58.9	58.9	2	5	2
62	75	M	NDMM	Bone marrow	20	TP53	P72R	250061	72	494	97.1	97.1	2	5	2
63	62	M	NDMM	Bone marrow	40	TP53	P72R	250061	72	576	97.4	97.4	1	1	1
64	73	F	NDMM	Bone marrow	50	KDR	R962C		962	4451	45.9	45.9	1	4	2
64	73	F	NDMM	Bone marrow	50	KDR	Q472H	149673	472	666	98.4	98.4	1	4	2
64	73	F	NDMM	Bone marrow	50	TP53	P72R	250061	72	466	52.5	52.5	1	4	2
65	75	F	NDMM	Bone marrow	100	KDR	Q472H	149673	472	832	41.5	41.5	1	1	2
65	75	F	NDMM	Bone marrow	100	MET	R988C	1666978	988	1955	50.8	50.8	1	1	2
65	75	F	NDMM	Bone marrow	100	TP53	P72R	250061	72	543	98.6	98.6	1	1	2
66	78	M	NDMM	Soft tissue	100	FGFR3	NA	1539830, 724	NA	539	51.8	51.8	2	8	2
66	78	M	NDMM	Soft tissue	100	KIT	M541L	28026	541	3498	100	100	2	8	2
66	78	M	NDMM	Soft tissue	100	KRAS	G12D	1135366, 521	12	1050	71	71	2	8	2
66	78	M	NDMM	Soft tissue	100	TP53	P72R	250061	72	335	54.7	54.7	2	8	2
67	31	M	NDMM	Bone marrow	60	KDR	Q472H	149673	472	958	48	48	3	6	2
67	31	M	NDMM	Bone marrow	60	KIT	M541L	28026	541	2464	51.7	51.7	3	6	2
67	31	M	NDMM	Bone marrow	60	TP53	P72R	250061	72	751	51	51	3	6	2
68	47	M	NDMM	Bone marrow	80	KDR	Q472H	149673	472	1248	50.7	50.7	0	0	2
68	47	M	NDMM	Bone marrow	80	NRAS	G13R	569	13	1902	16.6	16.6	0	0	2
68	47	M	NDMM	Bone marrow	80	TP53	P72R	250061	72	866	96.3	96.3	0	0	2
69	69	F	NDMM	Bone marrow	70	KIT	M541L	28026	541	3691	52.2	52.2	1	1	1
69	69	F	NDMM	Bone marrow	70	KRAS	G13D	1140132, 532	13	1798	5.8	8.3	1	1	1
69	69	F	NDMM	Bone marrow	70	NRAS	Q61R	584	61	4007	3.5	5	1	1	1
69	69	F	NDMM	Bone marrow	70	TP53	P72R	250061	72	586	98.8	98.8	1	1	1
70	60	M	NDMM	Bone marrow	100	ATM	F858L	21826	858	7833	49.9	49.9	3	7	3
70	60	M	NDMM	Bone marrow	100	TP53	P72R	250061	72	731	52.5	52.5	3	7	3
71	71	M	rrMM	Bone marrow	100	NRAS	Q61H	585	61	4568	42.5	42.5	1	4	2
71	71	M	rrMM	Bone marrow	100	TP53	P72R	250061	72	682	53.1	53.1	1	4	2
72	65	M	NDMM	Bone marrow	10	KDR	Q472H	149673	472	439	57.9	57.9	2	8	2
72	65	M	NDMM	Bone marrow	10	NRAS	G12S	580	12	4632	2	20	2	8	2
72	65	M	NDMM	Bone marrow	10	TP53	R283C	10911	283	206	47.1	47.1	2	8	2

72	65	M	NDMM	Bone marrow	10	TP53	P72R	250061	72	313	98.8	98.8	2	8	2
72	65	M	NDMM	Bone marrow	10	VHL	T100A	17856	100	4045	47.6	47.6	2	8	2
73	39	F	NDMM	Bone marrow	70	KDR	Q472H	149673	472	646	100	100	0	0	2
73	39	F	NDMM	Bone marrow	70	TP53	P72R	250061	72	847	62.5	62.5	0	0	2
74	71	F	NDMM	Bone marrow	15	MET	N375S	710	375	644	44.2	44.2	0	0	3
74	71	F	NDMM	Bone marrow	15	TP53	P72R	250061	72	665	50.7	50.7	0	0	3
75	50	F	NDMM	Bone marrow	75	TP53	P72R	250061	72	458	58.8	58.8	1	1	2
76	61	M	NDMM	Bone marrow	40	TP53	P72R	250061	72	334	100	100	1	3	2
77	59	M	NDMM	Bone marrow	80	TP53	P72R	250061	72	401	98.3	98.3	1	2	2
78	58	F	NDMM	Bone marrow	30	BRAF	V600E	476	600	1190	3.2	10.7	2	2	2
78	58	F	NDMM	Bone marrow	30	NRAS	Q61K	580	61	4203	15.7	15.7	2	2	2
78	58	F	NDMM	Bone marrow	30	TP53	P72R	250061	72	312	49.4	49.4	2	2	2
79	57	F	NDMM	Bone marrow	70	KDR	Q472H	149673	472	1263	52.2	52.2	2	4	1
79	57	F	NDMM	Bone marrow	70	KRAS	G12V	1140133, 520	12	1288	16.3	16.3	2	4	1
79	57	F	NDMM	Bone marrow	70	TP53	P72R	250061	72	723	98	98	2	4	1
80	85	F	NDMM	Bone marrow	100	KDR	Q472H	149673	472	912	51.3	51.3	3	7	3
80	85	F	NDMM	Bone marrow	100	KRAS	G12A	522, 25881	12	1343	2	2	3	7	3
80	85	F	NDMM	Bone marrow	100	PIK3CA	I391M	328028	391	721	100	100	3	7	3
80	85	F	NDMM	Bone marrow	100	TP53	P72R	250061	72	505	96.6	96.6	3	7	3
81	58	M	rrMM	Bone marrow	70	KDR	Q472H	149673	472	1085	45.4	45.4	0	0	2
81	58	M	rrMM	Bone marrow	70	TP53	P72R	250061	72	331	48	48	0	0	2
82	64	M	NDMM	Bone marrow	40	ATM	F858L	21826	858	2968	44.1	44.1	1	3	2
82	64	M	NDMM	Bone marrow	40	NRAS	Q61K	580	61	2302	5.4	13.5	1	3	2
83	52	F	NDMM	Bone marrow	90	KIT	M541L	28026	541	4357	52.9	52.9	2	8	3
83	52	F	NDMM	Bone marrow	90	NRAS	Y64D	1666991	64	6354	3.9	4.3	2	8	3
83	52	F	NDMM	Bone marrow	90	TP53	P72R	250061	72	579	100	100	2	8	3
84	47	F	NDMM	Bone marrow	90	BRAF	V600E	476	600	706	21.3	21.3	3	6	2
84	47	F	NDMM	Bone marrow	90	TP53	P72R	250061	72	262	98.9	98.9	3	6	2
85	57	F	NDMM	Bone marrow	90	KDR	Q472H	149673	472	368	53.9	53.9	0	0	3
85	57	F	NDMM	Bone marrow	90	MET	T1010I	707	1010	1054	47.3	47.3	0	0	3
85	57	F	NDMM	Bone marrow	90	TP53	P72R	250061	72	291	99	99	0	0	3
86	64	M	NDMM	Bone marrow	90	KRAS	G12S	1152506, 394409, 517	12	1606	14.6	14.6	1	2	3
86	64	M	NDMM	Bone marrow	90	TP53	P72R	250061	72	717	98.9	98.9	1	2	3
87	44	M	rrMM	Bone marrow	60	TP53	R282W	10704, 1636702, 99925	282	179	40	40	0	0	3
87	44	M	rrMM	Bone marrow	60	TP53	P72R	250061	72	167	93.5	93.5	0	0	3
88	80	M	NDMM	Bone marrow	60	ABL1	NA		NA	1575	50	50	3	1	2
88	80	M	NDMM	Bone marrow	60	KDR	Q472H	149673	472	306	3.6	6	3	1	2
88	80	M	NDMM	Bone marrow	60	MET	N375S	710	375	243	47.8	47.8	3	1	2
88	80	M	NDMM	Bone marrow	60	TP53	P72R	250061	72	293	95.4	95.4	3	1	2
89	60	F	rrMM	Bone marrow	10	ATM	F858L	21826	858	2069	47.5	47.5	0	0	2
89	60	F	rrMM	Bone marrow	10	KDR	Q472H	149673	472	780	49.8	49.8	0	0	2

89	60	F	rrMM	Bone marrow	10	PIK3CA	I391M	328028	391	504	57.2	57.2	0	0	2
89	60	F	rrMM	Bone marrow	10	TP53	P72R	250061	72	396	48.5	48.5	0	0	2
90	53	M	NDMM	Soft tissue	100	KRAS	Q61R	1158660, 552	61	2883	20.7	20.7	1	1	3
90	53	M	NDMM	Soft tissue	100	TP53	P72R	250061	72	271	39.8	39.8	1	1	3
91	76	M	NDMM	Bone marrow	20	BRAF	V600E	476	600	1468	9.6	48	2	3	2
91	76	M	NDMM	Bone marrow	20	TP53	P72R	250061	72	846	48.9	48.9	2	3	2
92	70	F	NDMM	Bone marrow	80	KRAS	G12V	1140133, 520	12	1711	15	15	0	0	2
93	57	M	rrMM	Bone marrow	10	TP53	P72R	250061	72	1051	98.8	98.8	0	0	2
94	65	M	NDMM	Bone marrow	10	TP53	P72R	250061	72	700	96.5	96.5	0	0	2
95	61	M	NDMM	Bone marrow	5	TP53	P72R	250061	72	418	97.7	97.7	3	10	3
96	36	M	NDMM	Bone marrow	10	PIK3CA	I391M	328028	391	863	60.2	60.2	0	0	3
96	36	M	NDMM	Bone marrow	10	TP53	P72R	250061	72	978	96.9	96.9	0	0	3
97	51	M	NDMM	Bone marrow	80	KRAS	G12C	1140136, 516	12	2872	29	29	2	8	2
97	51	M	NDMM	Bone marrow	80	TP53	P72R	250061	72	556	57.6	57.6	2	8	2
98	75	M	rrMM	Bone marrow	50	AKT1	R174C		174	4353	45.3	45.3	0	0	2
98	75	M	rrMM	Bone marrow	50	KDR	Q472H	149673	472	519	47.1	47.1	0	0	2
98	75	M	rrMM	Bone marrow	50	TP53	P72R	250061	72	393	95.9	95.9	0	0	2
99	76	F	NDMM	Bone marrow	60	NRAS	Q61R	584	61	4888	9.6	16	0	0	2
99	76	F	NDMM	Bone marrow	60	TP53	P72R	250061	72	549	95.9	95.9	0	0	2
100	52	M	NDMM	Bone marrow	40	TP53	P72R	250061	72	337	96.6	96.6	2	3	2
101	63	M	NDMM	Bone marrow	10	KDR	Q472H	149673	472	122	53.3	53.3	0	0	2
101	63	M	NDMM	Bone marrow	10	TP53	P72R	250061	72	109	67.2	67.2	0	0	2
102	76	M	NDMM	Bone marrow	15	JAK2	V617F	12600	617	1854	6.9	46	0	0	3
102	76	M	NDMM	Bone marrow	15	KDR	Q472H	149673	472	1142	50.6	50.6	0	0	3
102	76	M	NDMM	Bone marrow	15	KIT	M541L	28026	541	3223	52.3	52.3	0	0	3
102	76	M	NDMM	Bone marrow	15	TP53	P72R	250061	72	667	49.6	49.6	0	0	3
103	68	M	NDMM	Bone marrow	40	NRAS	G13D	573	13	1663	10.5	10.5	2	2	1
103	68	M	NDMM	Bone marrow	40	TP53	P72R	250061	72	804	50.2	50.2	2	2	1
104	74	F	NDMM	Bone marrow	80	FGFR1	NA		NA	555	21.9	21.9	2	3	2
104	74	F	NDMM	Bone marrow	80	KIT	M541L	28026	541	3259	49.2	49.2	2	3	2
104	74	F	NDMM	Bone marrow	80	NRAS	Q61K	580	61	4216	13.1	13.1	2	3	2
104	74	F	NDMM	Bone marrow	80	TP53	P72R	250061	72	832	38.9	38.9	2	3	2
105	73	M	rrMM	Bone marrow	30	KIT	M541L	28026	541	3886	49.1	49.1	2	4	2
105	73	M	rrMM	Bone marrow	30	KRAS	Q61H	1135364, 554	61	2935	5.4	18	2	4	2
105	73	M	rrMM	Bone marrow	30	TP53	P72R	250061	72	739	49.4	49.4	2	4	2
106	56	F	NDMM	Bone marrow	80	KDR	Q472H	149673	472	1031	44	44	3	8	3
106	56	F	NDMM	Bone marrow	80	KRAS	G13V	1152504, 534, 537	13	1839	23.9	23.9	3	8	3
106	56	F	NDMM	Bone marrow	80	TP53	NA	301220, 301221, 301222, 43963	NA	3548	21.9	21.9	3	8	3
106	56	F	NDMM	Bone marrow	80	TP53	P72R	250061	72	894	96.9	96.9	3	8	3
107	64	M	NDMM	Bone marrow	30	FGFR1	NA		NA	489	28.8	28.8	0	0	3
107	64	M	NDMM	Bone marrow	30	FGFR1	NA		NA	497	12.8	12.8	0	0	3

107	64	M	NDMM	Bone marrow	30	TP53	P72R	250061	72	1099	97.5	97.5	0	0	3
108	67	F	rrMM	Bone marrow	90	KDR	Q472H	149673	472	1610	48.2	48.2	0	0	2
108	67	F	rrMM	Bone marrow	90	MET	N375S	710	375	918	47.5	47.5	0	0	2
108	67	F	rrMM	Bone marrow	90	PIK3CA	I391M	328028	391	1374	52.5	52.5	0	0	2
108	67	F	rrMM	Bone marrow	90	SRC	Q529*		529	3915	44	44	0	0	2
108	67	F	rrMM	Bone marrow	90	TP53	P72R	250061	72	1176	96.7	96.7	0	0	2
109	72	F	rrMM	Bone marrow	90	KDR	Q472H	149673	472	2754	34	34	2	4	2
109	72	F	rrMM	Bone marrow	90	KIT	M541L	28026	541	4672	38.4	38.4	2	4	2
110	61	M	NDMM	Bone marrow	20	TP53	P72R	250061	72	614	60.6	60.6	1	1	2
111	69	F	rrMM	Bone marrow	100	BRAF	D594G	467	594	1345	61.4	61.4	1	2	1
111	69	F	rrMM	Bone marrow	100	KDR	Q472H	149673	472	941	50.4	50.4	1	2	1
111	69	F	rrMM	Bone marrow	100	TP53	R273L	10779, 1640828, 318169	273	543	56.9	56.9	1	2	1
111	69	F	rrMM	Bone marrow	100	TP53	Y220D	11847	220	1492	40.4	40.4	1	2	1
111	69	F	rrMM	Bone marrow	100	TP53	P33R		33	657	59.8	59.8	1	2	1
112	56	M	NDMM	Bone marrow	80	KRAS	G12V	1140133, 520	12	1981	13.8	13.8	3	5	3
112	56	M	NDMM	Bone marrow	80	TP53	P72R	250061	72	665	97.9	97.9	3	5	3
113	66	F	NDMM	Bone marrow	100	KRAS	G12D	1135366, 521	12	797	45	45	3	8	2
113	66	F	NDMM	Bone marrow	100	MET	T1010I	707	1010	895	49.9	49.9	3	8	2
114	81	F	rrMM	Bone marrow	90	KDR	Q472H	149673	472	921	44.1	44.1	3	4	2
114	81	F	rrMM	Bone marrow	90	TP53	P72R	250061	72	640	71.4	71.4	3	4	2
115	57	M	NDMM	Bone marrow	90	TP53	R337C	11071, 117591	337	1305	40.1	40.1	0	0	3
115	57	M	NDMM	Bone marrow	90	TP53	NA	43848, 984954, 984956, 984957, 984958	NA	1918	11.7	11.7	0	0	3
115	57	M	NDMM	Bone marrow	90	TP53	P72R	250061	72	975	94.4	94.4	0	0	3
116	63	F	NDMM	Bone marrow	25	ATM	E2444K	1506625, 1506626	2444	1708	13.5	13.5	0	0	3
117	56	F	NDMM	Bone marrow	100	JAK3	V722I	34213	722	1472	47.3	47.3	0	0	2
117	56	F	NDMM	Bone marrow	100	KDR	Q472H	149673	472	1001	51.4	51.4	0	0	2
117	56	F	NDMM	Bone marrow	100	MET	T1010I	707	1010	1965	46.9	46.9	0	0	2
117	56	F	NDMM	Bone marrow	100	TP53	P33R		33	1257	53.7	53.7	0	0	2
118	68	M	NDMM	Bone marrow	20	KIT	M541L	28026	541	4199	48.4	48.4	1	1	1
119	78	F	rrMM	Bone marrow	50	KDR	N1216T		1216	2847	49.4	49.4	0	0	2
119	78	F	rrMM	Bone marrow	50	KDR	Q472H	149673	472	1326	52.3	52.3	0	0	2
119	78	F	rrMM	Bone marrow	50	KIT	M541L	28026	541	2614	49.8	49.8	0	0	2
119	78	F	rrMM	Bone marrow	50	TP53	L265Q	44926	265	543	3.7	7.4	0	0	2
119	78	F	rrMM	Bone marrow	50	TP53	P72R	250061	72	699	43.7	43.7	0	0	2
120	45	M	NDMM	Bone marrow	80	KDR	Q472H	149673	472	52	52.8	52.8	1	7	2
120	45	M	NDMM	Bone marrow	80	NRAS	Q61R	584	61	2901	19.3	19.3	1	7	2
120	45	M	NDMM	Bone marrow	80	TP53	P72R	250061	72	58	64.1	64.1	1	7	2
121	62	M	NDMM	Bone marrow	50	KIT	M541L	28026	541	3340	54.7	54.7	2	1	2
121	62	M	NDMM	Bone marrow	50	KRAS	Q61H	1135364, 554	61	2473	10.6	10.6	2	1	2
121	62	M	NDMM	Bone marrow	50	TP53	P72R	250061	72	1210	97.5	97.5	2	1	2
122	35	M	NDMM	Bone marrow	40	JAK3	V722I	34213	722	336	54.9	54.9	3	9	3

122	35	M	NDMM	Bone marrow	40	MET	R988C	1666978	988	443	47.7	47.7	3	9	3
122	35	M	NDMM	Bone marrow	40	TP53	P72R	250061	72	63	100	100	3	9	3
123	63	F	NDMM	Bone marrow	20	KDR	Q472H	149673	472	1256	45.2	45.2	0	0	2
123	63	F	NDMM	Bone marrow	20	KIT	M541L	28026	541	2892	50.4	50.4	0	0	2
123	63	F	NDMM	Bone marrow	20	KRAS	K117N	1562192, 28519	117	516	7.9	39.5	0	0	2
123	63	F	NDMM	Bone marrow	20	TP53	P72R	250061	72	738	100	100	0	0	2
124	56	M	NDMM	Bone marrow	60	NRAS	Q61K	580	61	4369	11.6	11.6	0	0	2
125	76	M	NDMM	Bone marrow	90	TP53	P72R	250061	72	1062	98.2	98.2	3	3	2
126	69	M	NDMM	Bone marrow	80	KDR	Q472H	149673	472	821	50.8	50.8	1	4	2
126	69	M	NDMM	Bone marrow	80	KRAS	Q61H	1135364, 554	61	2432	10.9	10.9	1	4	2
127	73	F	NDMM	Bone marrow	80	CDKN2A	unknown	87424	unknown	793	6	7.5	1	2	2
127	73	F	NDMM	Bone marrow	80	KIT	M541L	28026	541	5891	51.9	51.9	1	2	2
127	73	F	NDMM	Bone marrow	80	KRAS	G12V	1140133, 520	12	1219	16.7	16.7	1	2	2
127	73	F	NDMM	Bone marrow	80	TP53	P72R	250061	72	211	71.7	71.7	1	2	2
128	80	F	NDMM	Bone marrow	40	KDR	Q472H	149673	472	700	95.4	95.4	1	1	2
128	80	F	NDMM	Bone marrow	40	KRAS	D119H		119	516	4.8	12	1	1	2
128	80	F	NDMM	Bone marrow	40	TP53	P72R	250061	72	697	57.3	57.3	1	1	2
129	76	F	NDMM	Bone marrow	90	FGFR3	R248C	1133721, 714	248	2643	20.4	20.4	0	0	3
129	76	F	NDMM	Bone marrow	90	TP53	P72R	250061	72	819	97.4	97.4	0	0	3
130	77	M	rrMM	Soft tissue	100	NRAS	Q61H	585	61	5141	65	65	3	8	3
131	60	F	rrMM	Bone marrow	60	No_mutation		No Mutations	NA	NA	NA	NA	0	0	3
132	64	F	rrMM	Bone marrow	90	KRAS	L19F	12703	19	5137	41	41	0	0	3
132	64	F	rrMM	Bone marrow	90	TP53	L155F		155	2249	35	35	0	0	3
132	64	F	rrMM	Bone marrow	90	TP53	M246V	43555	246	1365	50	50	0	0	3
133	60	F	rrMM	Cells	85	NRAS	Q61H	586	61	1893	50	50	0	0	3
133	60	F	rrMM	Cells	85	RB1	R320*	891	320	967	100	100	0	0	3
134	71	M	rrMM	Cells	85	KRAS	G12D	521	12	2247	43	43	2	8	3
135	65	M	rrMM	Cells	91.8	IDH1	R132H	28746	132	1330	45	45	1	3	3
135	65	M	rrMM	Cells	91.8	KRAS	G12V	520	12	948	97	97	1	3	3
136	61	F	rrMM	Cells	85	No_mutation		No Mutations	NA	NA	NA	NA	2	2	3
137	44	M	rrMM	Cells	85	TP53	I195T	11089	195	1974	14	14	2	8	3
138	75	M	rrMM	Bone marrow	70	NRAS	Q61R	584	61	3517	27	27	2	9	3
138	75	M	rrMM	Bone marrow	70	SMARCB1	M366_R369del		366	857	29	29	2	9	3
138	75	M	rrMM	Bone marrow	70	TP53	L137Q		137	971	10	14.3	2	9	3
139	73	F	rrMM	Cells	85	NRAS	G12R	561	12	2063	46	46	2	3	3
140	65	F	rrMM	Cells	85	NRAS	Q61R	584	61	1423	26	26	1	5	3
141	76	M	rrMM	Cells	85	PIK3CA	I391M		391	2105	55	55	3	9	3
142	65	F	rrMM	Cells	85	NRAS	G13V	574	13	1414	27	27	1	1	3
143	64	F	rrMM	Cells	85	ATM	Splice Site		NA	1565	47	47	3	6	3
143	64	F	rrMM	Cells	85	KRAS	K117N	28519	117	2205	55	55	3	6	3
144	76	M	rrMM	Cells	85	No_mutation		No Mutations	NA	NA	NA	NA	0	0	3

145	71	M	rrMM	Cells	85	KRAS	Q61H	554	61	3370	19	19	2	7	3
146	65	M	rrMM	Cells	85	RB1	366fs*10		366	1425	31	31	3	2	3
147	66	M	rrMM	Cells	85	No_mutation		No Mutations	NA	NA	NA	NA	3	1	3
148	73	M	rrMM	Cells	30.9	ERBB4	R232*		232	1254	32	32	0	0	3
148	73	M	rrMM	Cells	30.9	KRAS	Q61H	554	61	4146	28	28	0	0	3
148	73	M	rrMM	Cells	30.9	TP53	R175H	10648	175	3464	42	42	0	0	3
149	85	F	rrMM	Cells	91.3	KRAS	G12D	521	12	2694	48	48	2	8	3
150	67	M	rrMM	Cells	44.4	KRAS	G12A	522	12	2701	19	19	1	1	3
151	65	F	rrMM	Cells	86.7	BRAF	D594N	27639	594	1991	34	34	2	9	3
151	65	F	rrMM	Cells	86.7	PTPN11	G503R	14259	503	2146	40	40	2	9	3
152	76	M	rrMM	Cells	99.5	BRAF	V600E	476	600	2350	31	31	2	3	3
153	73	F	rrMM	Cells	83.1	KRAS	G13D	532	13	2658	34	34	2	1	3
154	61	F	rrMM	Cells	62.5	No_mutation		No Mutations	NA	NA	NA	NA	1	1	3
155	49	M	rrMM	Bone marrow	90	KRAS	G12V	520	12	3454	25	25	2	5	3
156	74	M	rrMM	Bone marrow	80	NRAS	Q61H	585	61	3059	25	25	1	1	3
157	69	F	rrMM	Cells	81.3	NRAS	Q61K	580	61	1069	39	39	2	5	3
158	67	M	rrMM	Cells	87.3	NRAS	G13D	573	13	3791	59	59	2	8	3
158	67	M	rrMM	Cells	87.3	STK11	Y156H		156	1145	34	34	2	8	3
159	47	M	rrMM	Cells	87.9	TP53	R273C	10659	273	1416	81	81	0	0	3
160	65	M	rrMM	Cells	88.1	ERBB4	F297fs*2		297	618	28	28	1	1	3
160	65	M	rrMM	Cells	88.1	KRAS	Q61H	554	61	2594	44	44	1	1	3
160	65	M	rrMM	Cells	88.1	TP53	R280S	44233	280	2086	38	38	1	1	3
161	57	M	rrMM	Cells	98.4	FGFR3	R248C	714	248	1095	5	5.1	2	2	3
161	57	M	rrMM	Cells	98.4	KRAS	Q61H	555	61	2033	39	39	2	2	3
162	78	M	rrMM	Cells	85	BRAF	V600E	476	600	1611	41	41	2	8	3
163	52	F	rrMM	Cells	98.3	KRAS	G12D	521	12	1718	54	54	2	3	3
164	59	F	rrMM	Cells	85	BRAF	G469R	457	469	1680	15	15	0	0	3
165	54	M	rrMM	Cells	85.5	No_mutation		No Mutations	NA	NA	NA	NA	0	0	3
166	60	F	rrMM	Cells	99.2	NRAS	G13D	573	13	1930	15	15	1	1	3
167	47	F	rrMM	Cells	94.9	No_mutation		No Mutations	NA	NA	NA	NA	0	0	3
168	66	M	rrMM	Cells	85	IDH1	R132H	28746	132	3335	48	48	1	2	3
168	66	M	rrMM	Cells	85	KRAS	G12S	517	12	1832	47	47	1	2	3
169	71	M	rrMM	Cells	91.3	No_mutation		No Mutations	NA	NA	NA	NA	0	0	3
170	66	F	rrMM	Cells	85	No_mutation		No Mutations	NA	NA	NA	NA	3	7	3
171	60	F	rrMM	Cells	86.3	KRAS	A18V	3735050	18	2506	34	34	3	8	3
171	60	F	rrMM	Cells	86.3	TP53	R181H	10738	181	3063	6	7	3	8	3
172	58	F	rrMM	Cells	84	No_mutation		No Mutations	NA	NA	NA	NA	2	2	3
173	50	M	rrMM	Cells	87.3	NRAS	Q61R	584	61	2514	19	19	0	0	3
174	52	M	rrMM	Cells	77.3	NRAS	Q61R	584	61	2772	38	38	0	0	3
174	52	M	rrMM	Cells	77.3	TP53	K132R	11582	132	2762	6	7.8	0	0	3
175	61	M	rrMM	Cells	98.1	BRAF	D594N	27639	594	1726	12	12	1	1	3

175	61	M	rrMM	Cells	98.1	FGFR3	*807C		807	3670	31	31	1	1	3
175	61	M	rrMM	Cells	98.1	KRAS	I24N	87313	24	2498	18	18	1	1	3
176	65	F	rrMM	Cells	85	NRAS	G12D	564	12	2992	64	64	1	1	3
176	65	F	rrMM	Cells	85	TP53	P151T	121046	151	1499	73	73	1	1	3
177	52	F	rrMM	Cells	85	NRAS	Q61K	580	61	3328	43	43	1	1	3
178	62	F	rrMM	Cells	85	NRAS	G13R	569	13	1236	30	30	2	1	3
179	75	F	rrMM	Cells	85	KRAS	Q61R	552	61	5508	95	95	3	8	3
180	65	F	rrMM	Cells	97	NRAS	G13D	573	13	1122	93	93	3	5	3
