# Supplementary figures and images for: Molecular signaling in multiple myeloma: association of RAS/RAF mutations and MEK/ERK pathway activation
Source: Oncogenesis. 2017 May 15;6(5):e337–. doi: 10.1038/oncsis.2017.36 (PMC5523069; doi:10.1038/oncsis.2017.36)

Figure 1S

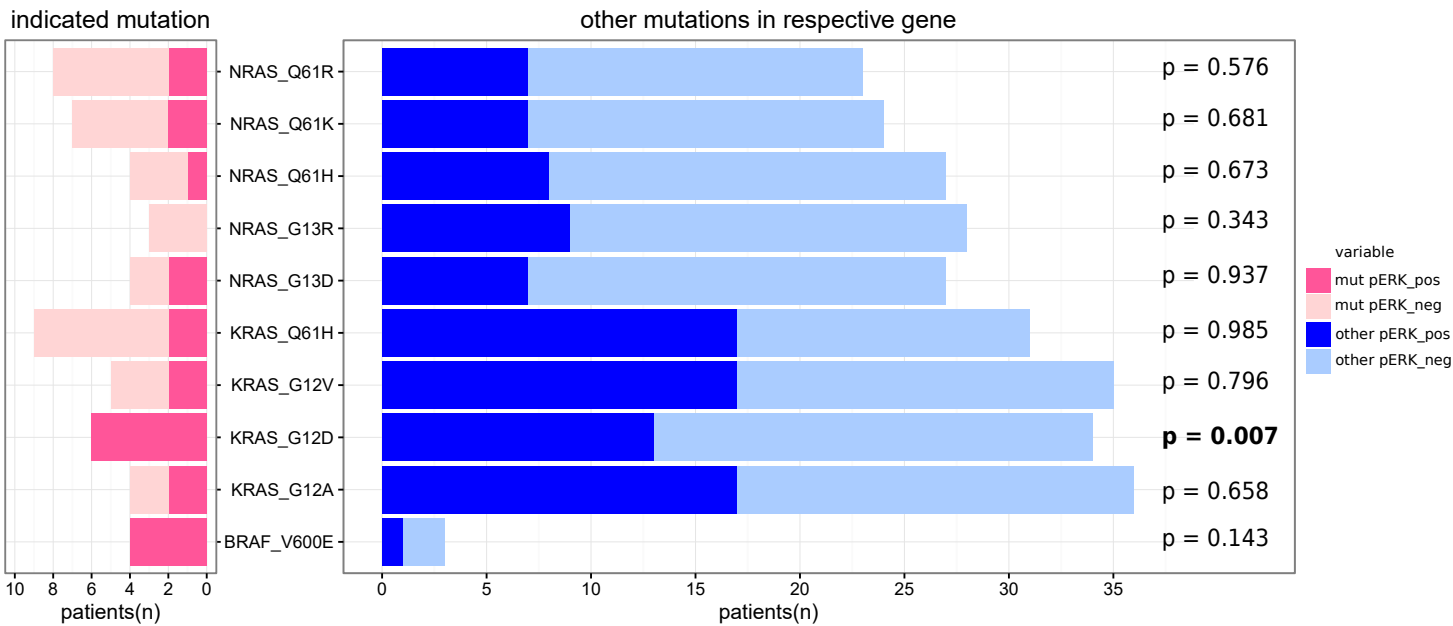

Supplement: Supplementary Figure 1 [file oncsis201736x3.pdf]

Figure S2

a.

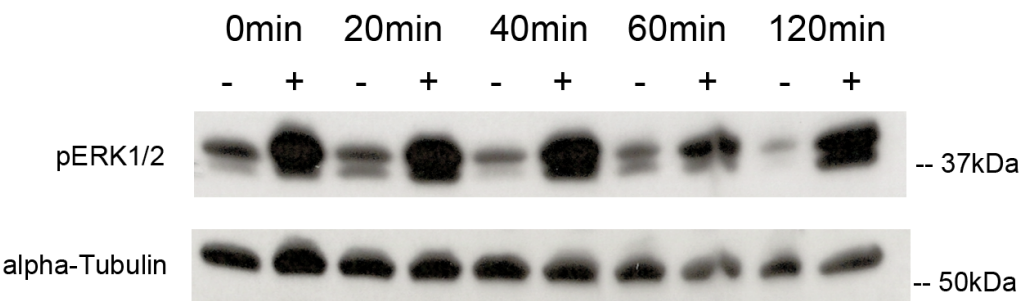

b.

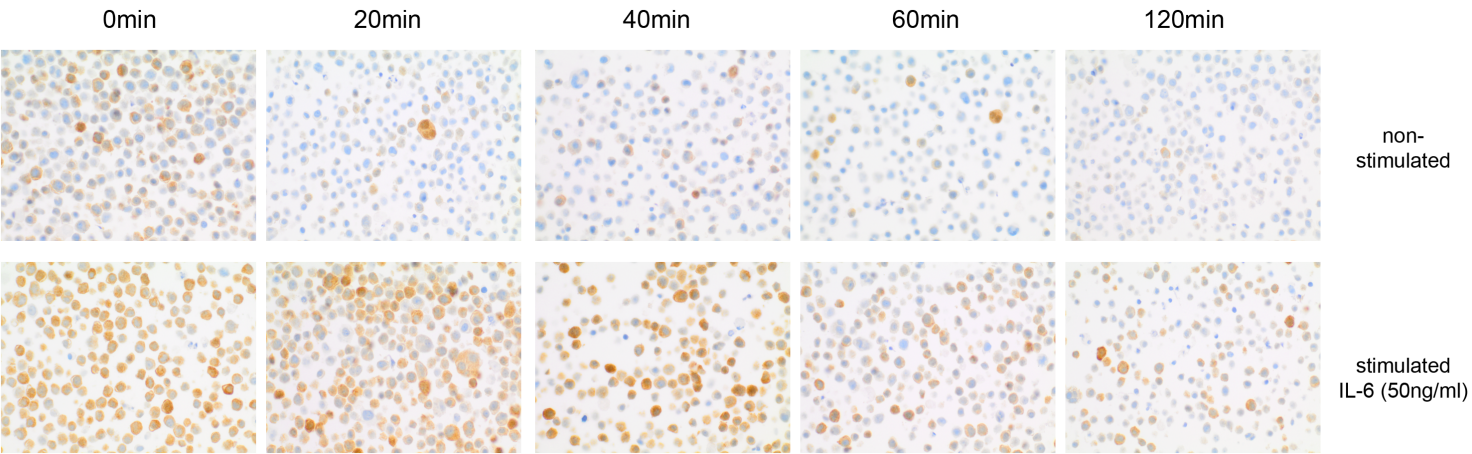

Supplement: Supplementary Figure 2 [file oncsis201736x4.pdf]
